# Mitogen-Activated Protein Kinase Signaling Regulates Proteoglycan Composition of Mast Cell Secretory Granules

**DOI:** 10.3389/fimmu.2018.01670

**Published:** 2018-07-19

**Authors:** Jun Mei Hu Frisk, Lena Kjellén, Fabio R. Melo, Helena Öhrvik, Gunnar Pejler

**Affiliations:** ^1^Department of Medical Biochemistry and Microbiology, Uppsala University, Uppsala, Sweden; ^2^Department of Anatomy, Physiology and Biochemistry, Swedish University of Agricultural Sciences, Uppsala, Sweden

**Keywords:** mast cells, mitogen-activated protein kinase, MEK1/2, proteoglycans, heparin, chondroitin sulfate, tryptase, serglycin

## Abstract

Mast cells (MCs) are characterized by an abundance of lysosome-like secretory granules filled with immunomodulatory compounds including histamine, cytokines, lysosomal hydrolases, MC-restricted proteases, and serglycin proteoglycans. The latter are essential for promoting the storage of other granule compounds and are built up of the serglycin core protein to which highly sulfated and thereby negatively charged glycosaminoglycan (GAG) side chains of heparin or chondroitin sulfate type are attached. In the search for mechanisms operating in regulating MC granule homeostasis, we here investigated the role of mitogen-activated protein kinase (MAPK) signaling. We show that inhibition of MEK1/2 (a MAPK kinase) leads to increased metachromatic staining of MC granules, indicative of increased proteoglycan content. Indeed, MEK1/2 inhibition caused a profound increase in the expression of the gene coding for the serglycin core protein and of genes coding for various enzymes involved in the biosynthesis/sulfation of the GAGs attached to the serglycin core protein. This was accompanied by corresponding increases in the levels of the respective GAGs. Deletion of the serglycin core protein abrogated the induction of enzymes operative in proteoglycan synthesis, indicating that availability of the serglycin proteoglycan core protein has a regulatory function impacting on the expression of the various serglycin-modifying enzymes. MEK1/2 inhibition also caused a substantial increase in the expression of granule-localized, proteoglycan-binding proteases. Altogether, this study identifies a novel role for MAPK signaling in regulating the content of secretory granules in MCs.

## Introduction

Mast cells (MCs) are crucial effector cells of the immune system, contributing to both the adaptive and innate arms of immune defense against external insults, such as pathogens and noxious substances including toxins from various venoms (1–4). In addition to their beneficial effects in host defense, MCs are also known to have detrimental functions during various pathologies of major impact for humans, including, in particular, allergic conditions, but also cancer, autoimmune diseases, atherosclerosis, and contact hypersensitivity (5, 6).

Mast cells are derived from the bone marrow, from which they egress as immature progenitors and then home to various tissues where they mature under the influence of local growth factors such as stem cell factor and IL-3 (7). In this process, they acquire an abundance of lysosome-like secretory granules, densely packed with numerous preformed bioactive substances, including histamine, cytokines/growth factors, lysosomal hydrolases, proteases [tryptase, chymase, carboxypeptidase A3 (CPA3)], and proteoglycans of serglycin type (8). Serglycin proteoglycans are critical for mediating the storage of various granule compounds, including proteases and bioactive amines (9), and are composed of the serglycin core protein to which glycosaminoglycans (GAGs) of either heparin/heparan sulfate (HS) or chondroitin sulfate (CS) type are attached. Heparin/HS is built up by repeating glucuronic acid (GlcUA)/iduronic acid (IdoUA)-*N*-acteylglucosamine (GlcNAc) disaccharide units whereas CS is built up by repeating GlcUA-*N*-acetylgalactosamine (GalNAc) disaccharide units (10). Both heparin/HS and CS are subject to extensive sulfation at various positions resulting in a high negative charge of the mature GAG, and the negative charge of the respective GAGs is essential for promoting the storage of granule components. When the MCs degranulate, a process that can be triggered by various stimuli including IgE receptor crosslinking, the preformed granule compounds are released to the external milieu where they can cause a powerful inflammatory reaction as exemplified by anaphylactic shock.

The processes leading to granule expulsion, i.e., degranulation, have been characterized extensively in numerous investigations [reviewed in Ref. (11–13)]. These investigations have uncovered a major role of various kinases, in particular Fyn, Lyn, and Syk in the initial phases of the signaling pathways downstream of antigen-mediated crosslinking of high-affinity IgE receptors (FcεRI). Activation of such kinases causes the phosphorylation and activation of adapters, such as Gab2, LAT1, and LAT2, leading to multiple effects including activation of PLC-γ and PI3-K. Downstream of these events is the influx of calcium leading to degranulation and activation of transcription factors including NF-κB and NFAT, but also activation of mitogen-activated protein kinase (MAPK) signaling. MAPK activation in MCs has been observed following various MC-activating regimens, including degranulation in response to FcεRI crosslinking (14–17), exposure to tumor cells (18), IL33-induced cytokine release (19), and TLR4 ligation (20). However, the potential role of MAPK signaling in the processes regulating MC granule homeostasis has not been investigated. Here, we addressed this issue. Intriguingly, we show that interference with MEK1/2, a MAPK kinase, has a major impact on the composition of the secretory granule proteoglycans and proteases. Thereby, MAPK signaling emerges as a novel player in the biogenesis of MC secretory granules.

## Materials and Methods

### Reagents

PD98059 (MEK inhibitor) was obtained from Sigma-Aldrich (Product number P215). U0126 (CAS109511-58-2) (MEK inhibitor) was purchased from EMD Millipore (Burlington, MA, USA). For both inhibitors, stock solutions were prepared in DMSO. The following antibodies were used: anti-ERK1/2, anti-P-ERK1/2, anti-P-JNK and anti-P-P38 [Cell Signaling Technology, anti-actin (I-19); Santa Cruz Biotechnology, Dallas, TX, USA], and anti-Mcpt6 antiserum (raised in rabbits). Monocloncal IgE anti-Dinitrophenyl (IgE anti-DNP) antibody was from Sigma (Stockholm, Sweden; product number: D8406). CellTiter-Blue Cell Viablity Assay was purchased from Promega-Invitrogen (Madison, WI, USA). The chromogenic substrate S-2288 was obtained from Chromogenix (Milan, Italy). May-Grünwald Eosine-methylene blue solution (product number: HX68862424) and Giemsa Azur-Eosine-methylene blue solution (product number: HX128350) were from Merck KGaA (Darmstadt, Germany). SYBR GreenER SuperMix and Rox reference dye were from Invitrogen (Carlsbad, CA, USA). Pierce phosphatase inhibitor was obtained from Thermo Fisher Scientific (Waltham, MA, USA) and protease inhibitor cocktail was from Roche Diagnostics.

### Animals and Cells

WT and serglycin knockout (Srgn^−/−^) (21) mice were on C57BL/6 genetic background. 4- or 5-month-old mice were used for experiments. All animal experiments were approved by the local ethical committee (no. C31/14; Uppsala djurförsöksetiska nämnd, Uppsala, Sweden). Bone marrow-derived MCs (BMMCs) were established as described in Ref. (22). The BMMCs were grown in media (10% FBS, 30% WEHI-3B-conditioned medium, 1% PEST, 1% l-glutamine, 58% Dulbecco’s modified Eagle’s medium) supplemented with 10 nM IL-3. The cell cultures were maintained at approximately 0.5–1 × 10^6^ cells/ml.

### Cell Viability

Cell viability was monitored with the CellTiter-Blue cell Viability assay. At each time point after addition of MEK inhibitor, 10 µl of cell viability reagent was mixed with 90 µl of cell suspension and incubated for 1 h at 37°C (5% CO_2_). A TECAN Infinite M200 plate reader was used for fluorescence scan measurements (560 nm for excitation and 590 nm for emission). Triplicate or quadruplicate determinations were performed. Three to four independent experiments were performed.

### Tryptase Activity

Twenty microliters of substrate S-2288 (10 mM) were mixed with 10 µl cell lysate and diluted with 90 µl H_2_O. Tryptase activity was monitored by reading the absorbance changes at 405 nm over 60 min using a microplate reader (Molecular Devices, Sunnyvale, CA, USA). Each measurement was performed in triplicates or quadruplicates, and the results represent the mean ± SD. Each experiment was repeated at least three times using different batches of cells.

### Degranulation Experiment

Cells were sensitized with 0.1 mg/ml IgE anti-DNP overnight. After IgE incubation, the cells were washed with PBS three times and resuspended in fresh BMMC medium and stimulated with DNP-human serum albumin (HSA) at 0.5 mg/ml for various time points. The cell pellets and supernatants were separated by centrifugation at 300 *g* for 10 min. β-hexosaminidase activity was measured as described in Ref. (22).

### May-Grünwald/Giemsa Staining

Fifty thousand BMMCs were used for each slide. The slides were air-dried and incubated with 100% May-Grünwald Eosine-methylene blue solution for 5 min and then 50% May-Grünwald Eosine-methylene blue solution for 1 min, followed by 15 min incubation in 2.5% Giemsa Azur Eosin-methylene solution and washing in H_2_O. The slides were dried before mounting. Experiments were repeated with five different batches of cells.

### Proteoglycan Analysis

PD98059-treated or non-treated cells were washed with PBS twice and then used for GAG analysis as described previously in Ref. (23, 24).

### Quantitative Real-Time PCR

RNA isolation kit was purchased from MACHEREY-NAGEL (Germany), and total RNA isolation from cell pellets was performed according to the instructions provided by the manufacturer. RNA purity and concentrations were assessed with a Nanodrop device. Two hundred nanograms of highly purified RNA (A260/280 > 1.95) were used for cDNA synthesis (the kit was from Bio-Rad, Solna, Sweden). Ten microliters of mixture (5 ml of SYBR GreenER SuperMix, 0.2 ml of primer mix, 3.6 ml of dH_2_O, 0.2 ml of Rox reference dye, and 1 ml of cDNA) were used for running quantitative real-time PCR (qPCR) with a 7900HT Fast Real-Time PCR System (Thermo Fisher Scientific). The following primers were used: Chst11 forward: CCA AAG TAT GTT GCA CCC AGT, Chst11 reverse: CTG GTC CCG TCT CAT CTG GT; Chst12 forward: CGC TAG GTC CGT CTC CCA G, Chst12 reverse: CAG ATA GAA GTG GGC GGT GC; Chst3 forward: CAT ATC CAG GGT CTC CGA CAA G, Chst3 reverse: CAA GAG AGA TGC ATT CTC CGA TAA G; Chst15 forward: GGC TTT TCA GGT CAC CTA CGA, Chst15 reverse: GAC ATT ATG GGT TCC TCG TTG A; Gapdh forward: CTC CCA CTC TTC CAC CTT CG, Gapdh reverse: CCA CCA CCC TGT TGC TGT AG; Ndst1 forward: CTG CAC TCC TGG ACC AAC CT, Ndst1 reverse: ACA GGG ATC CTG CCA AAG C; Ndst2 forward: GTG GCT GAT GTT GAG GCT TTG, Ndst2 reverse: ATC CTC CTC TTC TGT CCC GG; Hs6st1 forward: CAG CCA ACA CGT CTG AAC TG, Hs6st1 reverse: CTA GAC AAA GAC AAT TAG AAG ACA AC; Hs2st forward: CCA TAT CTC CCA GAT CGT GAC, Hs2st reverse: GTT ATA TGT TCT AAG GAC TCA GGC TC; Ext1 forward: GTG TAC CCG CAG CAG AAA GG, Ext1 reverse: GTA GAA CCT GGA GCC CTC GAT; Ext2 forward: CAA AAT CCG AGT TCC CCT GAA, Ext2 reverse: TCG ATT TCG TCG TAA GGG AAG; Cpa3 forward: TGA CAG GGA GAA GGT ATT CCG, Cpa3 reverse: CCA AGG TTG ACT GGA TGG TCT; Mcpt4 forward: GCA GTC TTC ACC CGA ATC TC, Mcpt4 reverse: CAG GAT GGA CAC ATG CTT TG; Mcpt6 forward: CAT TGA TAATGA CGA GCC TCT CC, Mcpt6 reverse: CAT CTC CCG TGT AGA GGC CAG; Srgn forward: GCA AGG TTA TCC TGC TCG GAG, Srgn reverse: GGT CAA ACT GTG GTC CCT TCT C. The program for qPCR was 50°C for 2 min, 95°C for 10 min, 95°C for 15 s, and 60°C for 1 min in 40 cycles and 95°C for 15 s, 60°C for 15 s, and 95°C for 15 s. GAPDH was used as a housekeeping gene. The relative amount of cDNA was determined in triplicates and calculated according to the 2^−ΔΔCT^ method (25). All data were derived from at least three independent experiments and are presented as the mean ± SD.

### Western Blot Analysis

Cell pellets were recovered after different treatments and were lysed with a modified RIPA buffer (50 mM Tris, 150 mM NaCl, 1% Nonidet P-40, 1% sodium deoxycholate, 0.1% SDS, and 1 mM EDTA) in the presence of phosphatase and protease inhibitors for 30–45 min on ice. The cell debris was removed by centrifugation at 14,000 rpm for 20 min at 4°C, and the supernatant was collected for protein analysis. Protein concentration was assessed using a Bicinchoninic Acid Kit (Sigma-Aldrich). Equal amounts of protein were used for western blot analysis, as described previously in Ref. (22).

### Statistical Analyses

All original data were analyzed by using the Microsoft Office Excel 2010 and GraphPad Prism software. Statistical comparisons were performed using a two-tailed *t*-test with the assumption of unequal variance. *P*-values less than 0.05 were considered significant. Each experiment was performed at least three times. Data are presented as the mean ± SD. Further details are given in the legends to the figures.

## Results

### MEK1/2 Inhibition Causes Increased Metachromatic Staining of MCs

**Figure 1:**
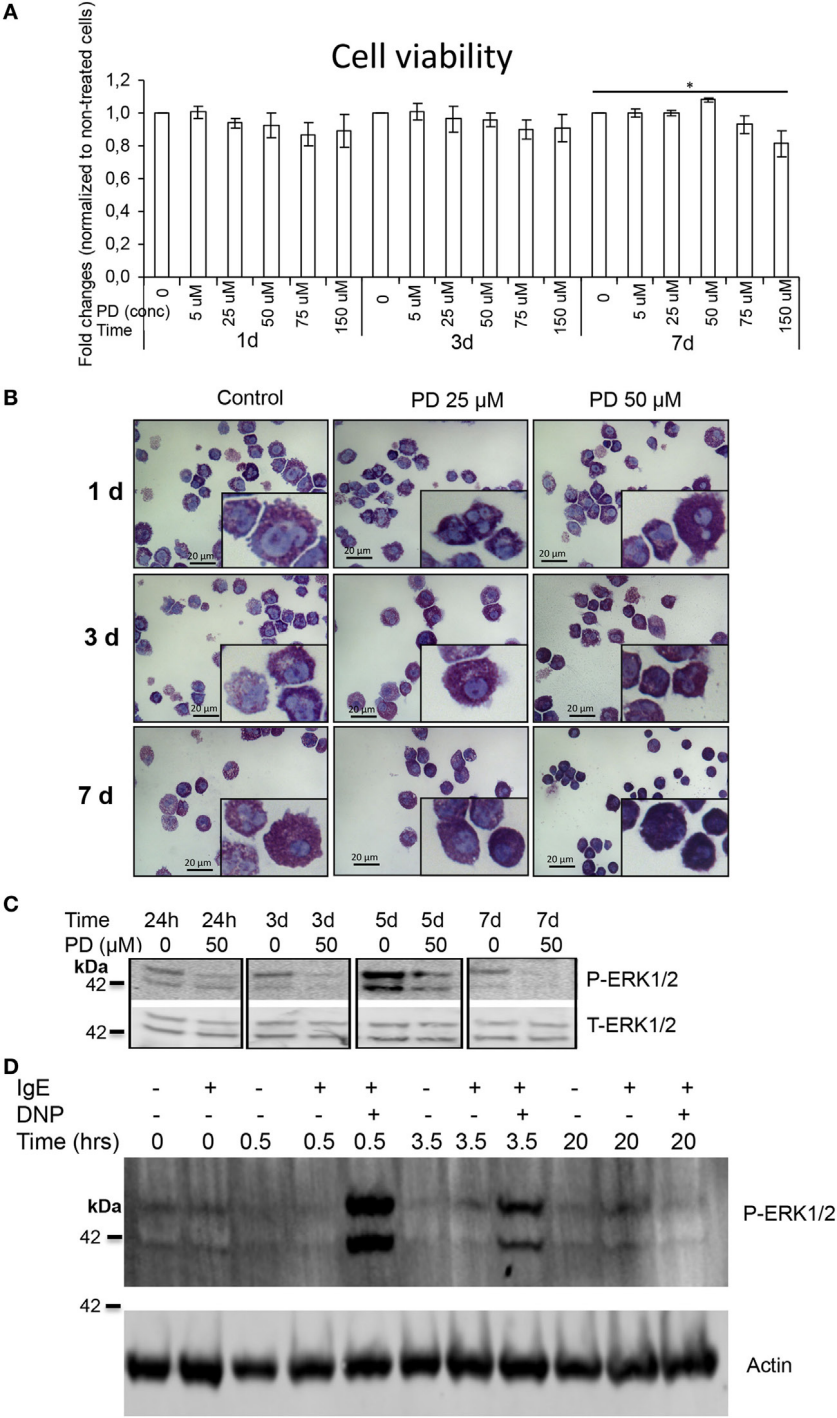
MEK1/2 inhibition induces granule maturation in mast cells (MCs). Bone marrow-derived mast cells (0.5–1.0 × 10^6^ cells/ml) were incubated with PD98059 (PD) at the concentrations and time periods indicated. **(A)** The viability of MCs after incubation with PD98059 was assessed using a CellTiter-Blue Cell Viablity Assay (*n* = 3). **(B)** After incubation with PD98059, cytospin slides were prepared and stained with May-Grünwald/Giemsa. The insets represent enlarged images of MCs with representative morphology. Note that treatment with PD98059 causes increased May-Grünwald/Giemsa staining intensity of granules. Original magnification: 40×. **(C)** Western blot analysis showing that the MEK1/2 inhibitor (PD98059; PD) causes reduced phosphorylation of ERK1/2 (P-ERK1/2), without affecting the total ERK1/2 levels (T-ERK1/2). **(D)** Western blot analysis of ERK1/2 phosphorylation after IgE receptor crosslinking. MCs (1 × 10^6^ cells) were incubated overnight either alone or with IgE anti-DNP (IgE) as indicated. Cells were then washed followed by the addition of DNP-HSA (DNP) to induce IgE receptor crosslinking as indicated. At the time points indicated, cells were recovered and analyzed for phosphorylated ERK1/2 (P-ERK1/2). Actin was used as loading control.

To address the possible role of MAPK signaling in regulating secretory granule homeostasis in MCs, we developed BMMCs from mice and exposed these populations to PD98059, a highly selective inhibitor of MEK1/2 (26), which are MAPK kinases that act by phosphorylation of ERK1/2. PD98059 was highly tolerated by BMMCs, showing no significant toxicity after incubation of BMMCs with PD98059 for up to 7 days at concentrations up to 75 µM (Figure 1A). As shown in Figure 1B and in agreement with observations made in a previous study in Ref. (27), incubation of BMMCs with PD98059 caused a profound increase in the metachromatic staining properties of the BMMCs. This effect was evident as early as after 24 h and was clearly visible also after long-term exposure to the MEK1/2 inhibitor (Figure 1B). Metachromatic staining of secretory granules is a hallmark feature of MCs, clearly distinguishing these cells from other cell types. Hence, these findings suggest that MAPK signaling has a role in regulating the homeostasis of the MC secretory granules. Increased metachromatic staining of BMMCs was also seen after incubation of the cells with an alternative MEK1/2 kinase inhibitor, U0126 (Figure S1 in Supplementary Material), supporting a role for MAPK signaling in regulating MC granule homeostasis. In contrast, incubation of MCs with inhibitors of p38 or JNK MAPKs did not affect the granular staining (Figure S2 in Supplementary Material), indicating that the observed increase in granular staining properties was specifically due to modulation of the MEK1/2–ERK1/2 axis.

To ascertain that PD98059 is efficient in dampening MAPK signaling in BMMCs and to monitor the durability of its effect in BMMCs, we performed western blot analysis to assess its effect on baseline ERK1/2 phosphorylation. Indeed, incubation of BMMCs with PD98059 resulted in decreased ERK1/2 phosphorylation without affecting the total levels of ERK1/2 (Figure 1C), and efficient inhibition was seen up to at least 7 days. In contrast, PD98059 did not have a major impact on the phosphorylation status of p38, and baseline JNK phosphorylation was undetectable in BMMCs (Figure S3 in Supplementary Material), indicating that PD98059 shows selectivity for the ERK1/2 pathway as opposed to other pathways of MAPK signaling.

To address whether MC activation initiates MAPK signaling, we subjected MCs to immunological activation by IgE receptor crosslinking and then examined effects on ERK1/2 phosphorylation. As seen in Figure 1D, ERK1/2 phosphorylation could be seen at baseline conditions, i.e., before inducing MC activation. However, when MCs were activated to degranulate by IgE receptor crosslinking, a dramatic increase in the phosphorylation of ERK1/2 was seen within 30 min of stimulation.

### MEK1/2 Inhibition Causes Increased Expression of Proteoglycan-Building Genes

**Figure 2:**
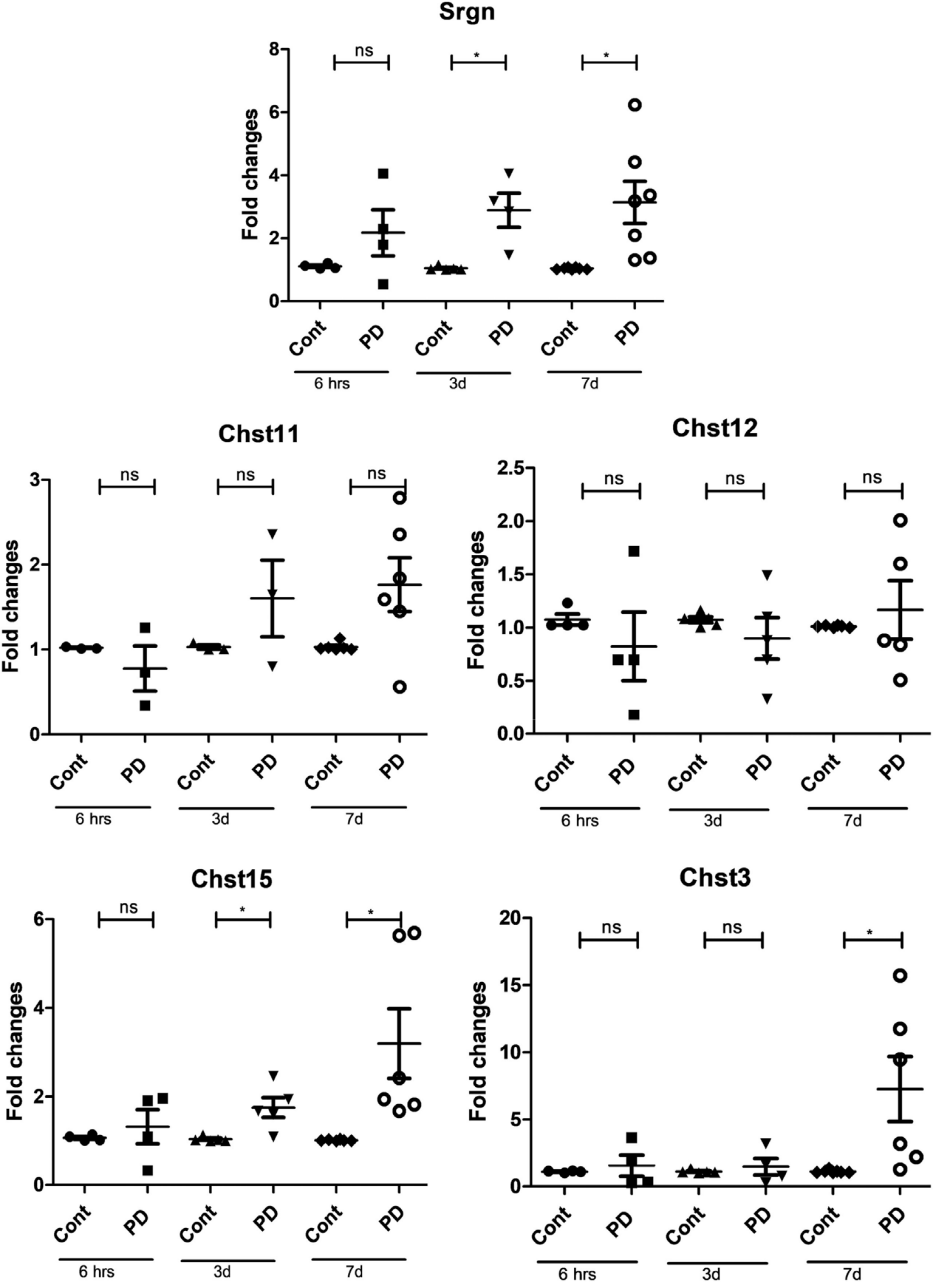
Inhibition of mitogen-activated protein kinase signaling causes increased expression of serglycin and genes involved in sulfation of chondroitin sulfate. Bone marrow-derived mast cells (0.5–1.0 × 10^6^ cells/ml) were incubated with 50 µM PD98059 (PD) for various time periods (6 h–7 days) as indicated. Total RNA was then prepared and analyzed by qPCR for the expression of serglycin (Srgn), Chst11, Chst12, Chst15, and Chst3 as indicated (*n* ≥ 4). Data were pooled from all experiments; at least four biological replicate experiments were performed, and each experiment was performed in triplicates (*p ≤ 0.05, **p ≤ 0.01, ***p ≤ 0.001).

The characteristic metachromatic staining properties of MCs is a result of binding of dye to highly negatively charged serglycin proteoglycans present in the MC secretory granules. As proof for this notion, deletion of the serglycin core protein, or deletion of enzymes responsible for sulfation of heparin chains attached to the serglycin core protein, causes abrogated metachromatic staining (21, 28). The increased metachromatic staining seen after MEK1/2 inhibition thus suggested an effect on proteoglycan content. To address this possibility, we first assessed whether PD98059 impacted on the expression of genes coding for enzymes involved in proteoglycan synthesis. As shown in Figure 2, long-term MEK1/2 inhibition resulted in a significant increase in the expression of the gene encoding the serglycin core protein (Srgn), as well as increased expression of some of the genes encoding enzymes that are involved in the biosynthesis of the GAGs (CS or heparin) that are attached to the serglycin core protein. The latter included Chst3, an enzyme catalyzing the 6-*O*-sulfation of GalNAc residues of CS, as well as Chst15, the enzyme catalyzing the 6-*O*-sulfation of 4-*O*-sulfated GalNAc residues. A trend of increased expression of Chst11 and Chst12 was also seen; the latter two enzymes catalyze the 4-*O*-sulfation of GalNAc residues in CS.

**Figure 3:**
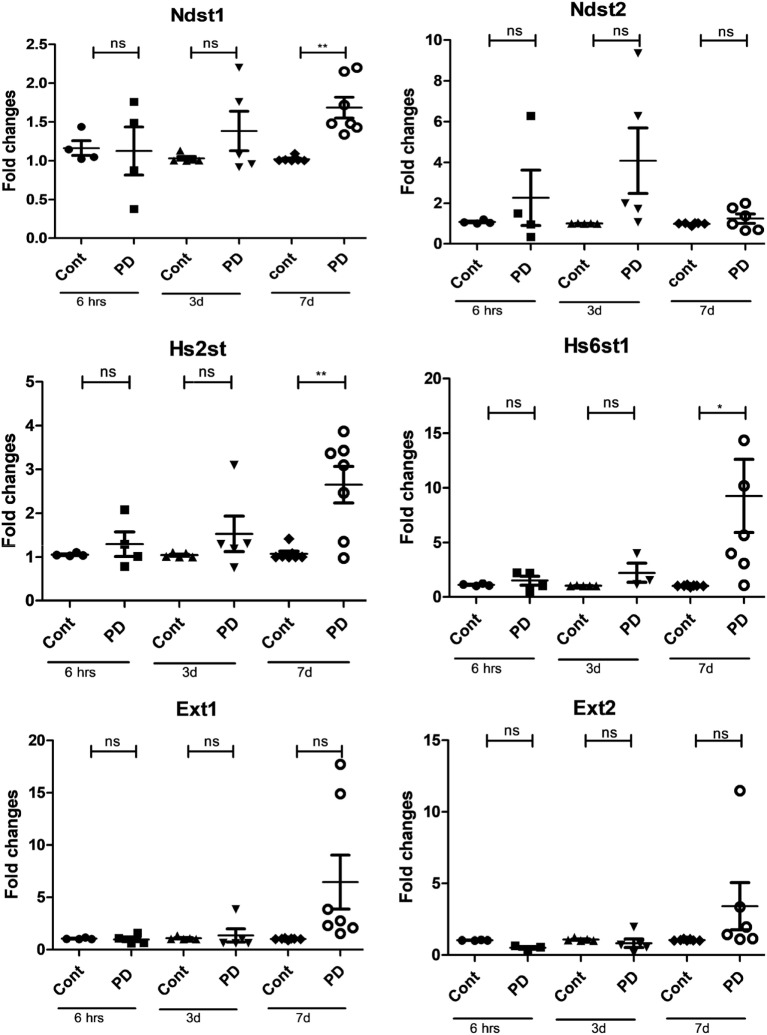
Mitogen-activated protein kinase signaling regulates the expression of genes involved in sulfation of heparin/heparan sulfate. Bone marrow-derived mast cells (0.5–1.0 × 10^6^ cells/ml) were incubated with 50 µM PD98059 (PD) for various time periods (6 h–7 days) as indicated. Total RNA was then prepared and analyzed by qPCR for the expression of Ndst1, Ndst2, Hs2st1, Hs6st1, Ext1, and Ext2 as indicated (*n* ≥ 4). Data were pooled from all experiments; at least four biological replicate experiments were performed, and each experiment was performed in triplicates (*p ≤ 0.05, **p ≤ 0.01, ***p ≤ 0.001).

Effects of long-term MEK1/2 inhibition on enzymes involved in heparin/HS synthesis were also seen. These included an induction of Ndst1, an enzyme catalyzing the *N*-deacetylation and subsequent *N*-sulfation of heparin/HS. Significant increases in the expression of the genes encoding Hs2Sst and Hs6st1 were also seen (Figure 3), the latter enzymes catalyzing the 2-*O*-sulfation of IdoUA residues and 6-*O*-sulfation of GlcNAc residues, respectively. In contrast, no significant effect was seen on the expression of Ext1 or Ext2, enzymes responsible for the elongation of heparin/HS chains.

### Increased Proteoglycan Content in MCs With Suppressed MEK1/2 Activity

**Figure 4:**
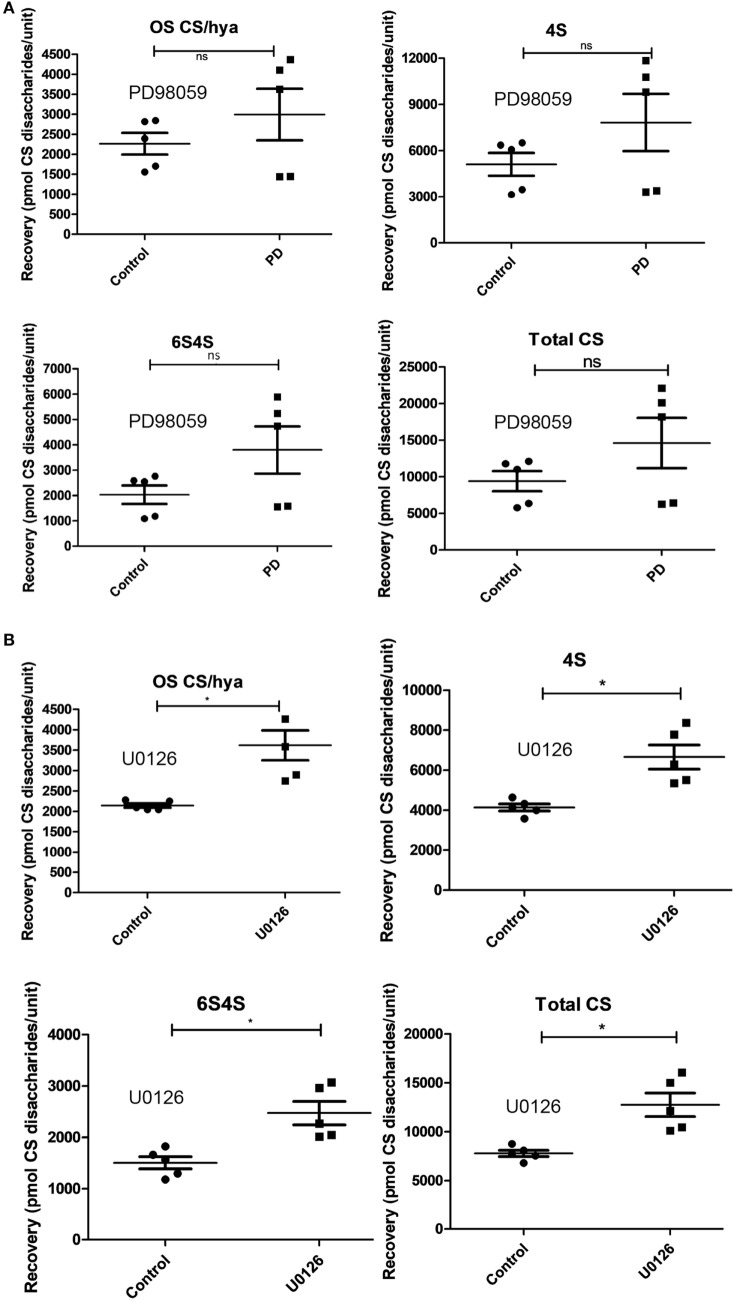
Effect of the inhibition of mitogen-activated protein kinase signaling on chondroitin sulfate in mast cells. Bone marrow-derived mast cells (0.5–1.0 × 10^6^ cells/ml) were incubated with **(A)** PD98059 (PD) at 50 µM for 7 days (*n* = 5; data were pooled from five biological replicates) or with **(B)** U0126 at 5 µM for 7 days (*n* = 5; data were pooled from five biological replicates). Glycosaminoglycan chains were then isolated, depolymerized to disaccharides, and analyzed by ion-pair-HPLC. The following chondroitin sulfate disaccharide species were identified and quantified: non-sulfated disaccharides of chondroitin sulfate or hyaluronan origin (OS CS/Hya); HexA-GalNAc $\left(4-O-{\text{SO}}_{3}^{-}\right)$ (4S); HexA-GalNAc $\left(4,6-\text{di}-O-{\text{SO}}_{3}^{-}\right)$ (4S6S); total chondroitin sulfate (Total CS) was also calculated. HexA, hexuronic acid (either glucuronic or iduronic acid); GalNAc, *N*-acetyl-galactosamine (*p ≤ 0.05, **p ≤ 0.01, ***p ≤ 0.001).

In order to assess whether the effect on expression of mRNAs encoding heparin/CS-synthesizing enzymes is translated to effects on the actual synthesized GAGs, we isolated GAGs from non-treated BMMCs and from BMMCs treated with MEK1/2 inhibitor (PD9805 or U0126) and determined their disaccharide composition by reverse-phase ion-pair-HPLC analysis (24). This analysis revealed a trend of increased amounts of non-sulfated (OS CS/Hya), 4-*O*-sulfated (4S), and highly sulfated CS disaccharides (6S4S) after treatment of BMMCs with PD98059, as well as a trend of increased total amounts of CS (Figure 4A). After treatment with U0126, a significant increase in the levels of non-sulfated (OS CS/Hya), 4-*O*-sulfated (4S) and highly sulfated CS disaccharides (6S4S) was seen, accompanied by a significant increase in the total amounts of CS (Figure 4B).

**Figure 5:**
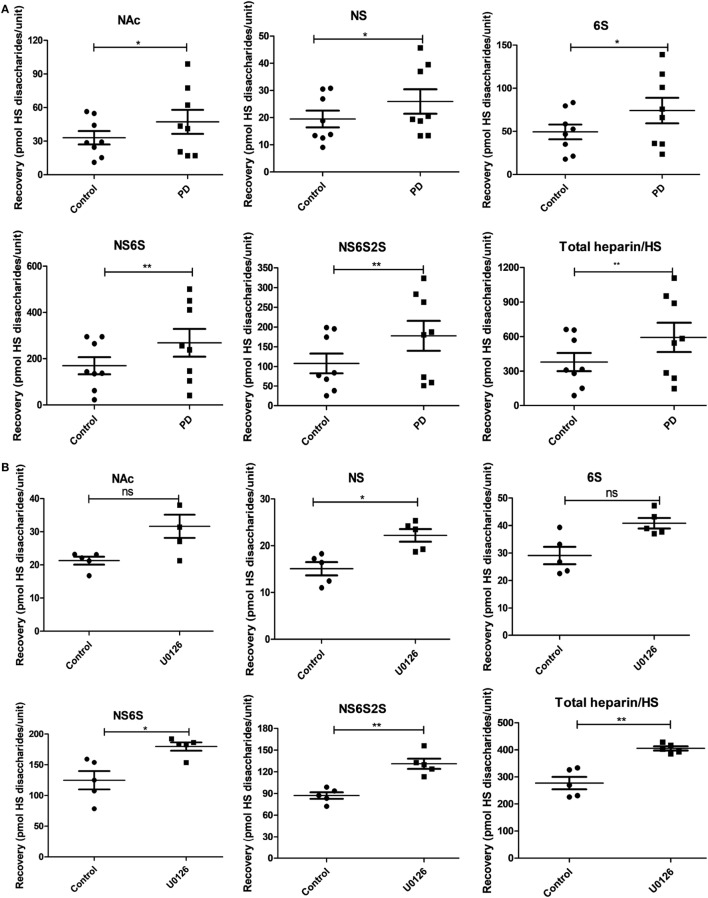
Effect of the inhibition of mitogen-activated protein kinase signaling on heparin/heparan sulfate (HS) in mast cells. Bone marrow-derived mast cells (0.5–1.0 × 10^6^ cells/mL) were incubated with **(A)** PD98059 (PD) at 50 µM for 7 days (n = 8; data were pooled from eight biological replicates) or with **(B)** U0126 at 5 µM for 7 days (n = 5; data were pooled from five biological replicates). Glycosaminoglycan chains were then isolated, depolymerized to disaccharides and analyzed by ion-pair-HPLC. The following heparin/HS disaccharide species were identified and quantified: HexA-GlcNAc (NAc); ${\text{HexA-GlcNSO}}_{3}^{-}$ (NS); $\text{HexA-GlcNAc}\left(\text{6-}O{\text{-SO}}_{3}^{-}\right)$ (6S); ${\text{HexA-GlcNSO}}_{3}^{-}\left(6-O-{\text{SO}}_{\text{3}}^{-}\right)$ (NS6S); $\text{HexA}\left(2-O-{\text{SO}}_{3}^{-}\right)-{\text{GlcNSO}}_{3}^{-}\left(6-O-{\text{SO}}_{3}^{-}\right)$ (NS6S2S). Total heparin/HS (Total heparin/HS) was also quantified. Abbreviations: HexA, hexuronic acid (either glucuronic acid or iduronic acid); GlcNAc, *N*-acetyl-glucosamine; GlcN, glucosamine.

As regards effects on heparin/HS, incubation of BMMCs with PD98059 caused significant increases in the levels of non-sulfated (NAc), N-sulfated (NS), 6-O-sulfated (6S), N-/6-O-disulfated (NS6S) and N-/2-O-/6-O-trisulfated (NS6S2S) disaccharides, as well as increased total amounts of heparin/HS (Figure 5A). Notably, the levels of CS were higher than those of heparin, both in non-treated and PD98059-treated cells (compare Figures 4A and 5A). The latter is thus in agreement with previous studies showing that CS is the major GAG species synthesized in BMMCs (10). After incubation of BMMCs with MEK1/2 inhibitor U0126, similar effects as for PD9805 treatment were seen (Figure 5B).

### Suppression of MEK1/2 Signaling Causes Upregulated Expression of MC Proteases

**Figure 6:**
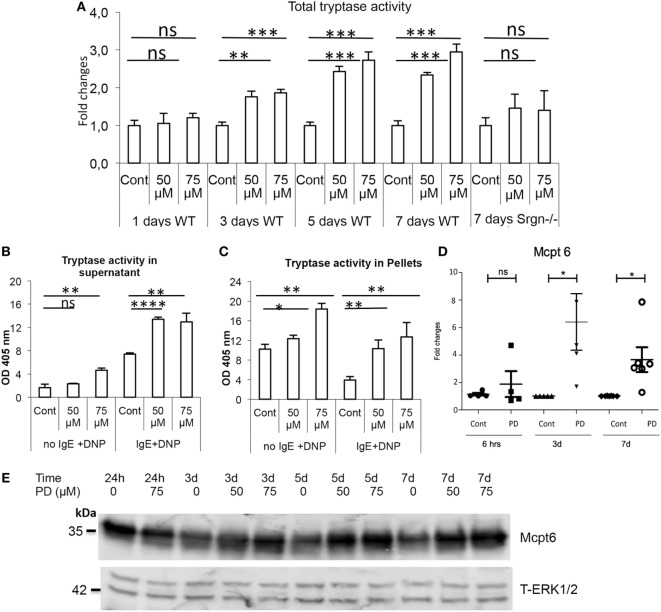
MEK1/2 inhibition in mast cells (MCs) causes increased tryptase activity and expression. Bone marrow-derived wild-type (WT) or serglycin^−/−^ (Srgn^−/−^) MCs (0.5–1.0 × 10^6^ cells/ml) were incubated with PD98059 (PD) at the concentrations and time periods indicated. **(A)** Total tryptase enzymatic activity in the cells was quantified by using a chromogenic substrate (S-2288). Note the robust increase in tryptase activity after long-term treatment with PD98059. **(B,C)** MCs were sensitized overnight as indicated by incubation with anti-DNP IgE, followed by IgE receptor crosslinking by addition of DNP-HSA. Next, tryptase activity in the supernatants **(B)** or in the residual cell pellets **(C)** was quantified. **(D)** MCs (0.5–1.0 × 10^6^ cells/ml) were incubated with PD98059 (50 µM) for the time periods indicated, followed by isolation of total RNA and quantification of mRNA encoding tryptase (Mcpt6) by qPCR (*n* ≥ 3). **(E)** Western blot analysis revealing increased levels of tryptase (Mcpt6) protein in MCs subjected to long-term treatment with PD98059 (50 µM). Total ERK1/2 was used as loading control (*n* = 6).

Previous studies have shown that serglycin proteoglycan is crucial for mediating the storage of the various proteases that are present in the MC granules (21). Since our data indicated that suppressed MAPK signaling causes increased synthesis of proteoglycans of serglycin type in MCs, we considered the possibility that this could be accompanied by alterations in the expression of serglycin-dependent MC proteases. To investigate if this was the case, we first quantified the levels of trypsin-like enzymatic activity, a measure of tryptase levels, in non-treated vs. PD98059-treated MCs. As depicted in Figure 6A, PD98059 treatment of BMMCs caused a robust increase in the amount of stored trypsin-like activity, a finding that is in agreement with an observation in a previous study in Ref. (27). This was seen after 3 days of culture and was more pronounced after 5–7 days. In contrast, no significant increase of tryptase content was seen after treatment of serglycin-null MCs with PD98059 (Figure 6A; right panel), in agreement with the known dependence of tryptase for serglycin for its storage in granules (21). Treatment of MCs with inhibitors of JNK or p38 MAPKs did not cause an increase in the levels of tryptase activity (data not shown).

To assess whether the increase in stored tryptase activity also was reflected by increased secretion of tryptase activity after MC activation, BMMCs were subjected to degranulation through IgE receptor crosslinking, followed by quantification of released tryptase activity. Indeed, BMMCs that had been treated with PD9805 for 7 days secreted substantially higher amounts of tryptase activity than did control cells (Figure 6B), and the residual tryptase activity in degranulated cells was also higher in the PD98059-treated cells (Figure 6C). Hence, MCs with suppressed MAPK signaling respond more vividly to IgE receptor crosslinking in terms of tryptase release.

In order to dissect the mechanisms underlying these latter findings, we asked whether the treatment of MCs with PD98059 caused an increase in the expression of tryptase mRNA. Indeed, long-term MEK1/2 inhibition in BMMCs caused a significant increase in the levels of mRNA coding for Mcpt6, the main tryptase expressed by murine MCs (Figure 6D). Further, as shown by western blot analysis, PD98059 treatment caused elevated levels of Mcpt6 at the protein level (Figure 6E).

We also assessed whether MEK1/2 inhibition affects proteases other than tryptase localized in the secretory granules. These analyses revealed that the levels of mRNA encoding Mcpt4 (a β-chymase) were significantly elevated, along with trend of upregulated Cpa3 expression (Figure S4 in Supplementary Material). Hence, interference with MEK1/2 has an overall stimulating effect on the expression of major proteases stored in the granules of MCs.

### Suppression of MEK1/2 Signaling Does Not Influence the Levels of β-Hexosaminidase in MCs

**Figure 7:**
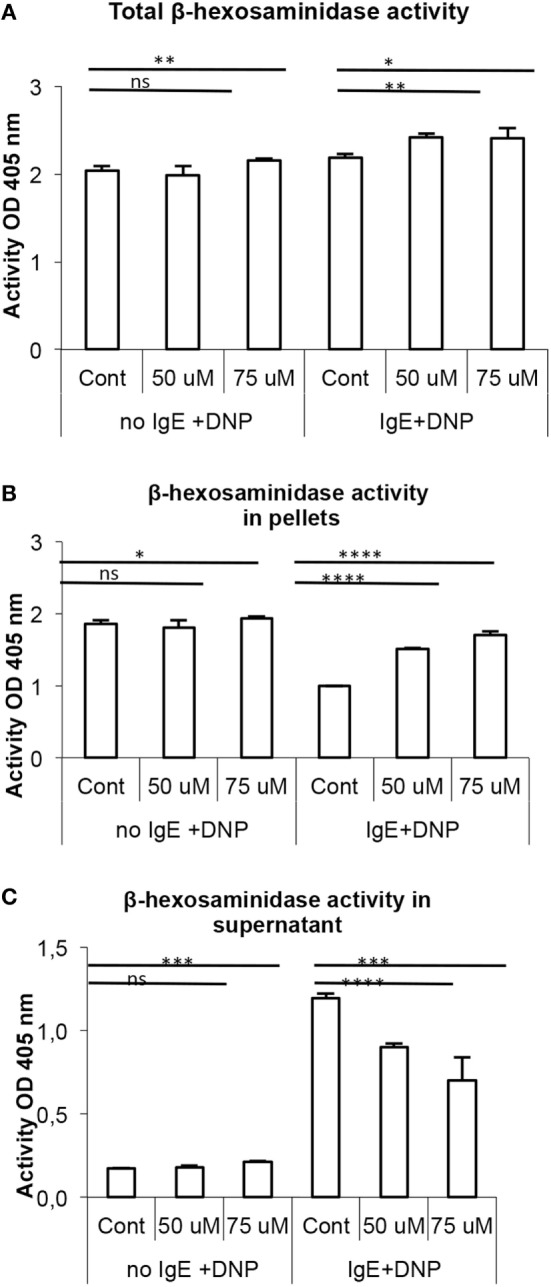
β-hexosaminidase storage is not affected by MEK1/2 inhibition. Bone marrow-derived mast cells (0.5–1.0 × 10^6^ cells/mL) were incubated for 7 days with PD98059 (PD) at the indicated concentrations. Cells were either non-sensitized or sensitized with anti-DNP IgE as indicated, followed by addition of DNP-HSA to induce IgE receptor crosslinking leading to degranulation. Cells were assayed for total β-hexosaminidase **(A)**, as well as for β-hexosaminidase activity in the supernatant **(B)** and in the residual cell pellets **(C)** (*p ≤ 0.05, **p ≤ 0.01, ***p ≤ 0.001, ****p ≤ 0.0001) (n = 3).

In addition to the serglycin proteoglycans and MC-restricted proteases, MC granules contain large amounts of lysosomal hydrolases, of which β-hexosaminidase is commonly used as marker for MC degranulation. To assess whether suppression of MEK1/2 also could affect the levels of this enzyme, we measured the levels of stored β-hexosaminidase activity in control vs. PD98059-treated BMMCs, and we also measured the amount of released β-hexosaminidase after subjecting MCs to IgE receptor crosslinking. In contrast to the robust effect of suppressed MEK1/2 activity on serglycin proteoglycans and on tryptase, PD98059 treatment was without effect on the baseline levels of stored β-hexosaminidase (Figure 7A). However, MEK1/2 inhibition caused an increased cellular retention of β-hexosaminidase following IgE receptor crosslinking (Figures 7B,C).

### Upregulated Expression of Proteoglycan-Building Genes in MCs With Suppressed MEK1/2 Signaling Depends on the Availability of the Serglycin Core Protein

**Figure 8:**
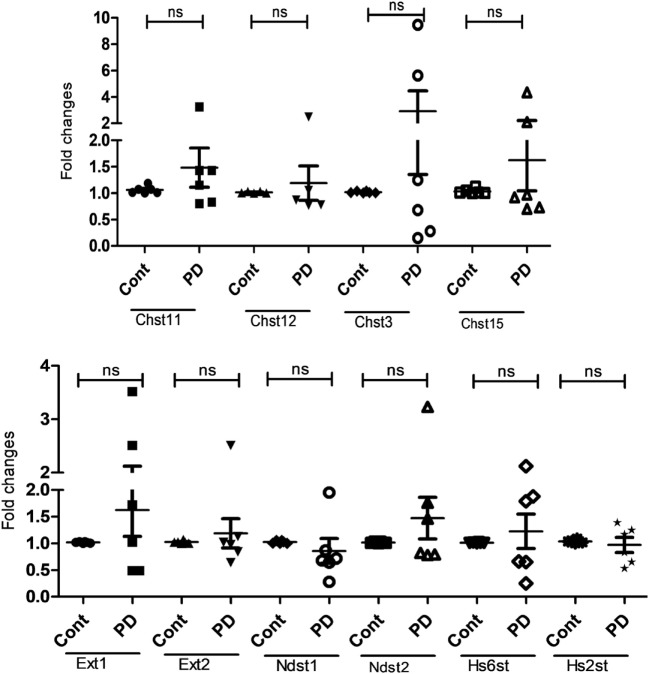
Upregulated expression of enzymes involved in chondroitin sulfate and heparin/heparan sulfate (HS) synthesis depends on the presence of the serglycin core protein. Bone marrow-derived mast cells (0.5–1.0 × 10^6^ cells/ml) were generated from serglycin^−/−^ mice and were incubated for 7 days with PD98059 (PD) (50 µM). Total RNA was then prepared and analyzed by qPCR for the expression of Chst11, Chst12, Chst3, Chst15, Ext1, Ext2, Ndst1, Ndst2, Hs6st1, Hs2st, as indicated (*n* ≥ 4; data were pooled from at least four biological replicate experiments). Abbreviation: ns, not significant.

Next, we aimed at providing insight into the mechanism underlying the effects of MAPK signaling on the MC proteoglycans and proteases. One potential scenario could be that the strongly upregulated proteoglycan synthesis seen after MEK1/2 inhibition could be dependent on the presence of positively charged binding partners to the serglycin proteoglycans. The MC-restricted proteases constitute major binding partners to serglycin (21, 28) and we, therefore, assessed if their absence has an impact on the regulation of enzymes involved in proteoglycan synthesis. However, the simultaneous absence of multiple MC-restricted proteases (Mcpt4, Mcpt5, Mcpt6, and Cpa3) (29) in MCs did not abrogate the upregulated expression of proteoglycan-related genes in response to PD98059 (data not shown). An alternative scenario could be that the upregulation of enzymes involved in proteoglycan synthesis is dependent on the availability of core protein onto which the GAG side chains are attached. To assess this possibility, we made use of BMMCs cultured from mice lacking the serglycin core protein, i.e., the common core protein onto which both CS and heparin chains are assembled. As seen in Figure 8, the absence of serglycin abrogated the PD98059-mediated induction of enzymes involved in proteoglycan synthesis. Hence, this suggests that the induction of enzymes involved in proteoglycan synthesis is dependent on the availability of the serglycin core protein.

## Discussion

Mast cell degranulation is a hallmark event in many pathological settings such as allergic reactions, and the signaling pathways and mechanisms involved in this process have accordingly been studied extensively (11–13). In contrast, there is to date relatively little insight into the mechanisms that operate during the reverse event, i.e., the biogenesis of the MC secretory granules (8, 30). In previous attempts to address this issue we have identified a key role for serglycin proteoglycan in maintaining granule homeostasis by promoting the storage of various granule-localized proteases, including tryptase (Mcpt6), CPA3, and selected chymases (Mcpt4 and Mcpt5) (21, 28), and also for promoting the storage of bioactive amines (31, 32).

In the process of MC degranulation, numerous signaling events are activated (see Introduction). However, the signaling events involved in the reverse process, i.e., the building up of granules stores, have received little attention. We show here that inhibition of MAPK signaling has a profound positive effect on granule content, suggesting that this signaling pathway has a hitherto unrecognized role in regulating the composition of the MC secretory granules. Our data provide evidence that interference with MEK1/2, a MAPKK, leads to a marked increase in the metachromatic staining of MC granules. The metachromatic staining of MC granules is strictly dependent on the presence of serglycin proteoglycan (21, 28), and these findings thus led us to hypothesize that MAPK signaling can regulate the synthesis of serglycin proteoglycan. Indeed, we show that MEK1/2 inhibition induced an elevated expression of the gene coding for the serglycin core protein (*Srgn*). In addition, genes coding for various enzymes involved in the sulfation of the heparin/HS and CS chains attached to the serglycin core protein were induced by interference with MEK1/2, including Chst15, Hs2st1, Hs6st1, and Ndst1.

In agreement with the effects seen at the gene expression level, we demonstrate that MEK1/2 inhibition affects biosynthesis of the granule GAGs. First, the interference with MAPK signaling caused an elevation of the total levels of GAGs of CS and heparin/HS type. This was reflected by increases of the respective disaccharide species that build up the respective GAGs. Altogether, these data, together with the effects seen at the gene expression level, indicate that MAPK signaling has a major impact on the levels of granule proteoglycans. Reduced MAPK signaling will thus result in a higher content of serglycin proteoglycans present in the MC granules, which will enhance the capacity of the granules to store positively charged compounds such as the various MC-restricted proteases and bioactive amines. Suppression of MAPK signaling may hence represent an important event in the processes leading to MC granule maturation.

We also show that interference with the MAPK pathway causes an increase in the levels of tryptase, a major granule constituent. Since tryptase storage is critically dependent on the highly sulfated serglycin proteoglycans in MCs, a likely explanation for this finding might be that the increase in proteoglycan content could cause a secondary positive effect on the storage of tryptase. If so, it may be expected that the increase in tryptase level upon PD98059 treatment is a result of increased retention of tryptase in the granules, without affecting tryptase gene expression. However, we found that the interference with MAPK signaling in fact induced the expression of Mcpt6, i.e., the gene encoding tryptase. Hence, the increase in the levels of tryptase is most likely due to increased *Mcpt6* expression accompanied by increased capacity of tryptase storage due to increased contents of granular proteoglycans. Moreover, the inhibition of MAPK signaling had positive effects on the expression of *Mcpt4*. These findings indicate that the MAPK pathway has a hitherto unrecognized role in promoting the expression of a panel of genes encoding compounds involved in building up the MC secretory granules.

The exact mechanism behind the upregulated expression of serglycin after interference with MAPK signaling is not entirely clear. However, a previous study suggested that MEK1/2 inhibition causes upregulated expression of the transcription factor MITF (27). MITF is a major transcription factor regulating MC development, having a key role in promoting multiple features of MCs including expression of MC proteases (33). MITF is known to regulate transcription through specific E-boxes (CANNTG motifs) (34), and an analysis of the serglycin gene reveals the presence of two CANNTG elements in the serglycin promoter (35). This suggests that serglycin expression can be enhanced by MITF. Intriguingly, previous studies have shown that MITF is phosphorylated by ERK1/2 and thereby directed to proteasomal degradation (36). Hence, a plausible scenario behind the increased serglycin expression in response to MEK1/2 inhibition is that inactivated MAPK signaling results in elevated MITF levels, thereby leading to increased expression of the serglycin gene.

An intriguing finding was that the induction of a number of genes involved in the biosynthesis of the heparin/HS and CS chains seen in wild-type (WT) cells subjected to MEK1/2 inhibition was abrogated in MCs lacking expression of the serglycin core protein. This suggests that there is crosstalk between the expression of the serglycin core protein and the expression of genes involved in serglycin core protein modification (by GAGs), such that the induction of enzymes involved in serglycin modification is critically dependent on the presence of the GAG acceptor, i.e., the serglycin core protein. However, the mechanism underlying this crosstalk remains to be identified.

Interestingly, inhibition of MAPK signaling did neither affect the levels of stored β-hexosaminidase, nor the ability of MCs to release β-hexosaminidase in response to IgE receptor crosslinking. Hence, the MAPK pathway specifically regulates the storage of selected granular compounds whereas others are unaffected. It is notable that β-hexosaminidase, in contrast to tryptase, chymase, and Cpa3, is not dependent on serglycin for storage (37), most likely explained by a lack of affinity for the anionic GAGs attached to the serglycin core protein. We may thus propose that MAPK signaling specifically affects the storage of serglycin and of those compounds that are stored dependently on serglycin.

Together, our findings identify a hitherto unrecognized role of MAPK signaling in regulating the contents of the MC secretory granules. MCs are known to have the ability to regenerate their granular stores following extensive degranulation, and that they thereby are capable of repeated degranulation (38–40). Hence, there is a need of efficient mechanisms to ensure that the granular contents are efficiently resynthesized in this process. Our findings suggest that suppression of MAPK signaling could represent a signaling mechanism operative in turning the MCs from a mode of degranulation to a regranulation mode. Notably, MC degranulation in response to, e.g., IgE receptor crosslinking is known to cause a strong but transient activation of MAPK signaling pathways, as shown both here and in numerous other studies (14–17). Hence, we may suggest that the decrease in MAPK pathway activation following the initial MAPK activation could represent a molecular switch to initiate efficient MC regranulation to ensure that the MCs become primed to carry out repeated degranulation.

## Ethics Statement

All animal experiments were approved by the local ethical committee (no. C31/14; Uppsala djurförsöksetiska nämnd, Uppsala, Sweden).

## Author Contributions

JF planned the study, performed and analyzed the experiments, interpreted the data, and contributed to the writing of the manuscript; LK contributed to the design of the study, interpreted data, and contributed to the writing of the manuscript; FM contributed to the experiments; HÖ contributed to the design of the study and contributed to the writing of the manuscript; GP designed the study, interpreted the data, and wrote the manuscript.

## Conflict of Interest Statement

The authors declare that the research was conducted in the absence of any commercial or financial relationships that could be construed as a potential conflict of interest.

## Supplementary Material

Figure S1 Mast cells (MCs) (1 × 10^6^ cells) were incubated with U0126 at the time periods and concentrations indicated. **(A)** Morphology of MCs as assessed by May-Grünwald/Giemsa staining of cytospin slides. **(B)** Cell viability as assessed by a CellTiter-Blue Cell Viablity Assay. **(C)** Tryptase activity of non-treated vs. U0126-treated cells, as determined by cleavage of a chromogenic tryptase substrate (S-2288).

Figure S2 JNK or P38 mitogen-activated protein kinase (MAPK) inhibition does not enhance granule maturation in mast cells (MCs). Bone marrow-derived mast cells (MCs) (0.5–1.0 × 10^6^ cells/ml) were incubated with SP600125 (JNK MAPK inhibitor) or SB203580 (p38 MAPK inhibitor) at the concentrations and time periods indicated. After incubation with the respective inhibitor, cytospin slides were prepared and stained with May-Grünwald/Giemsa. The insets represent enlarged images of MCs with representative morphology. Note that neither of the inhibitors caused increased May-Grünwald/Giemsa staining intensity of granules. Original magnification: 40×.

Figure S3 Mast cells (1 × 10^6^ cells) were left untreated or were treated with PD 98059 at the time periods and concentrations indicated. Cells were recovered and analyzed by western blotting for phosphorylation of P38 and JNK. Actin was used as loading control.

Figure S4 Mast cells (0.5–1.0 × 10^6^ cells/ml) were incubated with PD98059 (50 µM) for the time periods indicated, followed by isolation of total RNA and quantification of mRNA encoding Mcpt4 and Cpa3 by qPCR (*n* ≥ 3).

## Abbreviations

MC, mast cell; BMMC, bone marrow-derived mast cell; CPA, carboxypeptidase A; MAPK, mitogen-activated protein kinase; GAG, glycosamimnoglycan; HS, heparan sulfate; CS, chondroitin sulfate; GlcUA, glucuronic acid; IdoUA, iduronic acid; GlcNAc, N-acteylglucosamine; GalNAc, N-acetylgalactosamine.

## References

[1] Marshall JS. Mast-cell responses to pathogens. Nat Rev Immunol (2004) 4:787–99. 10.1038/nri1460 15459670

[2] Johnzon CF Rönnberg E Pejler G. The role of mast cells in bacterial infection. Am J Pathol (2016) 186:4–14. 10.1016/j.ajpath.2015.06.024 26477818

[3] Akahoshi M Song CH Piliponsky AM Metz M Guzzetta A Abrink M Mast cell chymase reduces the toxicity of Gila monster venom, scorpion venom, and vasoactive intestinal polypeptide in mice. J Clin Invest (2011) 121:4180–91. 10.1172/JCI46139 21926462 PMC3195461

[4] Metz M Piliponsky AM Chen CC Lammel V Abrink M Pejler G Mast cells can enhance resistance to snake and honeybee venoms. Science (2006) 313:526–30. 10.1126/science.1128877 16873664

[5] Voehringer D. Protective and pathological roles of mast cells and basophils. Nat Rev Immunol (2013) 13:362–75. 10.1038/nri3427 23558889

[6] Galli SJ Grimbaldeston M Tsai M. Immunomodulatory mast cells: negative, as well as positive, regulators of immunity. Nat Rev Immunol (2008) 8:478–86. 10.1038/nri2327 18483499 PMC2855166

[7] Gurish MF Austen KF. Developmental origin and functional specialization of mast cell subsets. Immunity (2012) 37:25–33. 10.1016/j.immuni.2012.07.003 22840841

[8] Wernersson S Pejler G Mast cell granules: armed for battle. Nat Rev Immunol (2014) 14:478–94. 10.1038/nri3690 24903914

[9] Kolset SO Pejler G. Serglycin: a structural and functional chameleon with wide impact on immune cells. J Immunol (2011) 187:4927–33. 10.4049/jimmunol.1100806 22049227

[10] Rönnberg E Melo FR Pejler G. Mast cell proteoglycans. J Histochem Cytochem (2012) 60:950–62. 10.1369/0022155412458927 22899859 PMC3527880

[11] Blank U Rivera J The ins and outs of IgE-dependent mast-cell exocytosis. Trends Immunol (2004) 25:266–73. 10.1016/j.it.2004.03.005 15099567

[12] Kinet JP. The high-affinity IgE receptor (Fc epsilon RI): from physiology to pathology. Annu Rev Immunol (1999) 17:931–72. 10.1146/annurev.immunol.17.1.931 10358778

[13] Alvarez-Errico D Lessmann E Rivera J. Adapters in the organization of mast cell signaling. Immunol Rev (2009) 232:195–217. 10.1111/j.1600-065X.2009.00834.x 19909365 PMC3018096

[14] Suzuki H Takei M Yanagida M Nakahata T Kawakami T Fukamachi H. Early and late events in Fc epsilon RI signal transduction in human cultured mast cells. J Immunol (1997) 159:5881–8. 9550384

[15] Song JS Gomez J Stancato LF Rivera J. Association of a p95 Vav-containing signaling complex with the FcepsilonRI gamma chain in the RBL-2H3 mast cell line. Evidence for a constitutive in vivo association of Vav with Grb2, Raf-1, and ERK2 in an active complex. J Biol Chem (1996) 271:26962–70. 10.1074/jbc.271.43.26962 8900182

[16] Song JS Haleem-Smith H Arudchandran R Gomez J Scott PM Mill JF Tyrosine phosphorylation of Vav stimulates IL-6 production in mast cells by a Rac/c-Jun N-terminal kinase-dependent pathway. J Immunol (1999) 163:802–10. 10395673

[17] Jabril-Cuenod B Zhang C Scharenberg AM Paolini R Numerof R Beaven MA Syk-dependent phosphorylation of Shc. A potential link between FcepsilonRI and the Ras/mitogen-activated protein kinase signaling pathway through SOS and Grb2. J Biol Chem (1996) 271:16268–72. 10.1074/jbc.271.27.16268 8663278

[18] Gorzalczany Y Akiva E Klein O Merimsky O Sagi-Eisenberg R. Mast cells are directly activated by contact with cancer cells by a mechanism involving autocrine formation of adenosine and autocrine/paracrine signaling of the adenosine A3 receptor. Cancer Lett (2017) 397:23–32. 10.1016/j.canlet.2017.03.026 28342985

[19] Drube S Kraft F Dudeck J Muller AL Weber F Gopfert C MK2/3 are pivotal for IL-33-induced and mast cell-dependent leukocyte recruitment and the resulting skin inflammation. J Immunol (2016) 197:3662–8. 10.4049/jimmunol.1600658 27694493

[20] Martin-Avila A Medina-Tamayo J Ibarra-Sanchez A Vazquez-Victorio G Castillo-Arellano JI Hernandez-Mondragon AC Protein tyrosine kinase fyn regulates TLR4-elicited responses on mast cells controlling the function of a PP2A-PKCalpha/beta signaling node leading to TNF secretion. J Immunol (2016) 196:5075–88. 10.4049/jimmunol.1501823 27183589

[21] Åbrink M Grujic M Pejler G. Serglycin is essential for maturation of mast cell secretory granule. J Biol Chem (2004) 279:40897–905. 10.1074/jbc.M405856200 15231821

[22] Rönnberg E Pejler G. Serglycin: the master of the mast cell. Methods Mol Biol (2012) 836:201–17. 10.1007/978-1-61779-498-8_14 22252637

[23] Ledin J Staatz W Li JP Gotte M Selleck S Kjellen L Heparan sulfate structure in mice with genetically modified heparan sulfate production. J Biol Chem (2004) 279:42732–41. 10.1074/jbc.M405382200 15292174

[24] Dagälv A Holmborn K Kjellén L Åbrink M. Lowered expression of heparan sulfate/heparin biosynthesis enzyme N-deacetylase/n-sulfotransferase 1 results in increased sulfation of mast cell heparin. J Biol Chem (2011) 286:44433–40. 10.1074/jbc.M111.303891 22049073 PMC3248000

[25] Livak KJ Schmittgen TD. Analysis of relative gene expression data using real-time quantitative PCR and the 2(-Delta Delta C(T)) method. Methods (2001) 25:402–8. 10.1006/meth.2001.1262 11846609

[26] Dudley DT Pang L Decker SJ Bridges AJ Saltiel AR. A synthetic inhibitor of the mitogen-activated protein kinase cascade. Proc Natl Acad Sci U S A (1995) 92:7686–9. 10.1073/pnas.92.17.7686 7644477 PMC41210

[27] Hu Frisk JM Kjellen L Kaler SG Pejler G Ohrvik H. Copper regulates maturation and expression of an MITF:Tryptase axis in mast cells. J Immunol (2017) 199:4132–41. 10.4049/jimmunol.1700786 29127151 PMC5728160

[28] Forsberg E Pejler G Ringvall M Lunderius C Tomasini-Johansson B Kusche-Gullberg M Abnormal mast cells in mice deficient in a heparin-synthesizing enzyme. Nature (1999) 400:773–6. 10.1038/23488 10466727

[29] Grujic M Calounova G Eriksson I Feyerabend T Rodewald HR Tchougounova E Distorted secretory granule composition in mast cells with multiple protease deficiency. J Immunol (2013) 191:3931–8. 10.4049/jimmunol.1301441 23975861

[30] Azouz NP Hammel I Sagi-Eisenberg R. Characterization of mast cell secretory granules and their cell biology. DNA Cell Biol (2014) 33:647–51. 10.1089/dna.2014.2543 24988214 PMC4180300

[31] Ringvall M Rönnberg E Wernersson S Duelli A Henningsson F Åbrink M Serotonin and histamine storage in mast cell secretory granules is dependent on serglycin proteoglycan. J Allergy Clin Immunol (2008) 121:1020–6. 10.1016/j.jaci.2007.11.031 18234316

[32] Rönnberg E Calounova G Pejler G. Mast cells express tyrosine hydroxylase and store dopamine in a serglycin-dependent manner. Biol Chem (2012) 393:107–12. 10.1515/BC-2011-220 22628305

[33] Morii E Oboki K Kataoka TR Igarashi K Kitamura Y. Interaction and cooperation of mi transcription factor (MITF) and myc-associated zinc-finger protein-related factor (MAZR) for transcription of mouse mast cell protease 6 gene. J Biol Chem (2002) 277:8566–71. 10.1074/jbc.M110392200 11751862

[34] Jippo T Morii E Tsujino K Tsujimura T Lee YM Kim DK Involvement of transcription factor encoded by the mouse mi locus (MITF) in expression of p75 receptor of nerve growth factor in cultured mast cells of mice. Blood (1997) 90:2601–8. 9326226

[35] Korpetinou A Skandalis SS Labropoulou VT Smirlaki G Noulas A Karamanos NK Serglycin: at the crossroad of inflammation and malignancy. Front Oncol (2014) 3:327. 10.3389/fonc.2013.00327 24455486 PMC3888995

[36] Turski ML Brady DC Kim HJ Kim BE Nose Y Counter CM A novel role for copper in Ras/mitogen-activated protein kinase signaling. Mol Cell Biol (2012) 32:1284–95. 10.1128/MCB.05722-11 22290441 PMC3302449

[37] Henningsson F Hergeth S Cortelius R Åbrink M Pejler G. A role for serglycin proteoglycan in granular retention and processing of mast cell secretory granule components. FEBS J (2006) 273:4901–12. 10.1111/j.1742-4658.2006.05489.x 17010166

[38] Burwen SJ. Recycling of mast cells following degranulation in vitro: an ultrastructural study. Tissue Cell (1982) 14:125–34. 10.1016/0040-8166(82)90012-X 7089960

[39] Dvorak AM Schleimer RP Lichtenstein LM. Morphologic mast cell cycles. Cell Immunol (1987) 105:199–204. 10.1016/0008-8749(87)90068-2 2434250

[40] Slutsky B Jarvis D Bibb P Feldberg RS Cochrane DE. Viability and recovery from degranulation of isolated rat peritoneal mast cells. Exp Cell Res (1987) 168:63–78. 10.1016/0014-4827(87)90416-2 2430821

