# Supplementary figures and images for: Mitogen-Activated Protein Kinase Signaling Regulates Proteoglycan Composition of Mast Cell Secretory Granules
Source: Front Immunol. 2018 Jul 19;9:1670. doi: 10.3389/fimmu.2018.01670 (PMC6060404; doi:10.3389/fimmu.2018.01670)

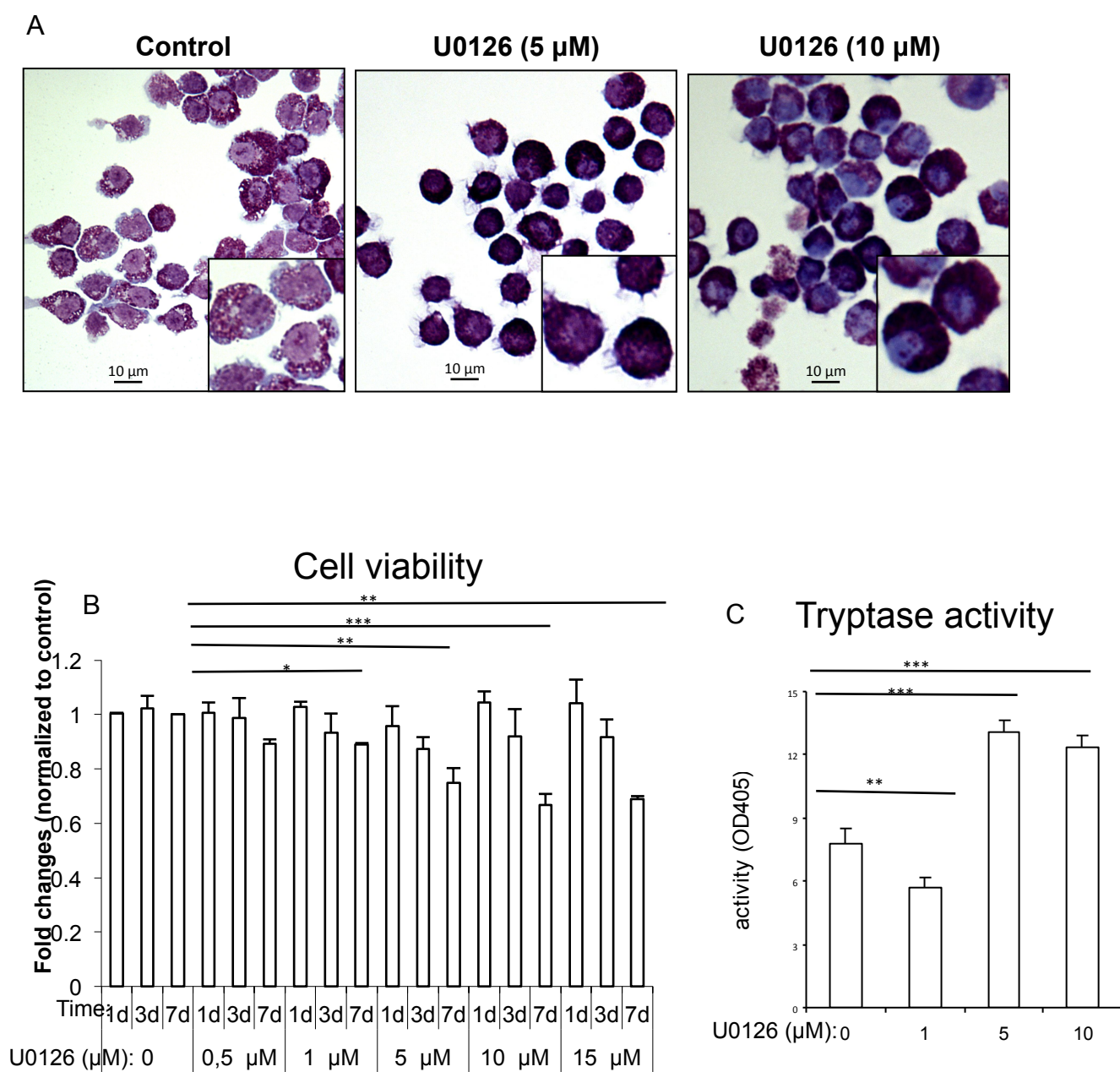

Suppl. Fig. 1

Supplement: Figure S1 — Mast cells (MCs) (1 × 10^6^ cells) were incubated with U0126 at the time periods and concentrations indicated. (A) Morphology of MCs as assessed by May-Grünwald/Giemsa staining of cytospin slides. (B) Cell viability as assessed by a CellTiter-Blue Cell Viablity Assay. (C) Tryptase activity of non-treated vs. U0126-treated cells, as determined by cleavage of a chromogenic tryptase substrate (S-2288). [file data_sheet_1.PDF]

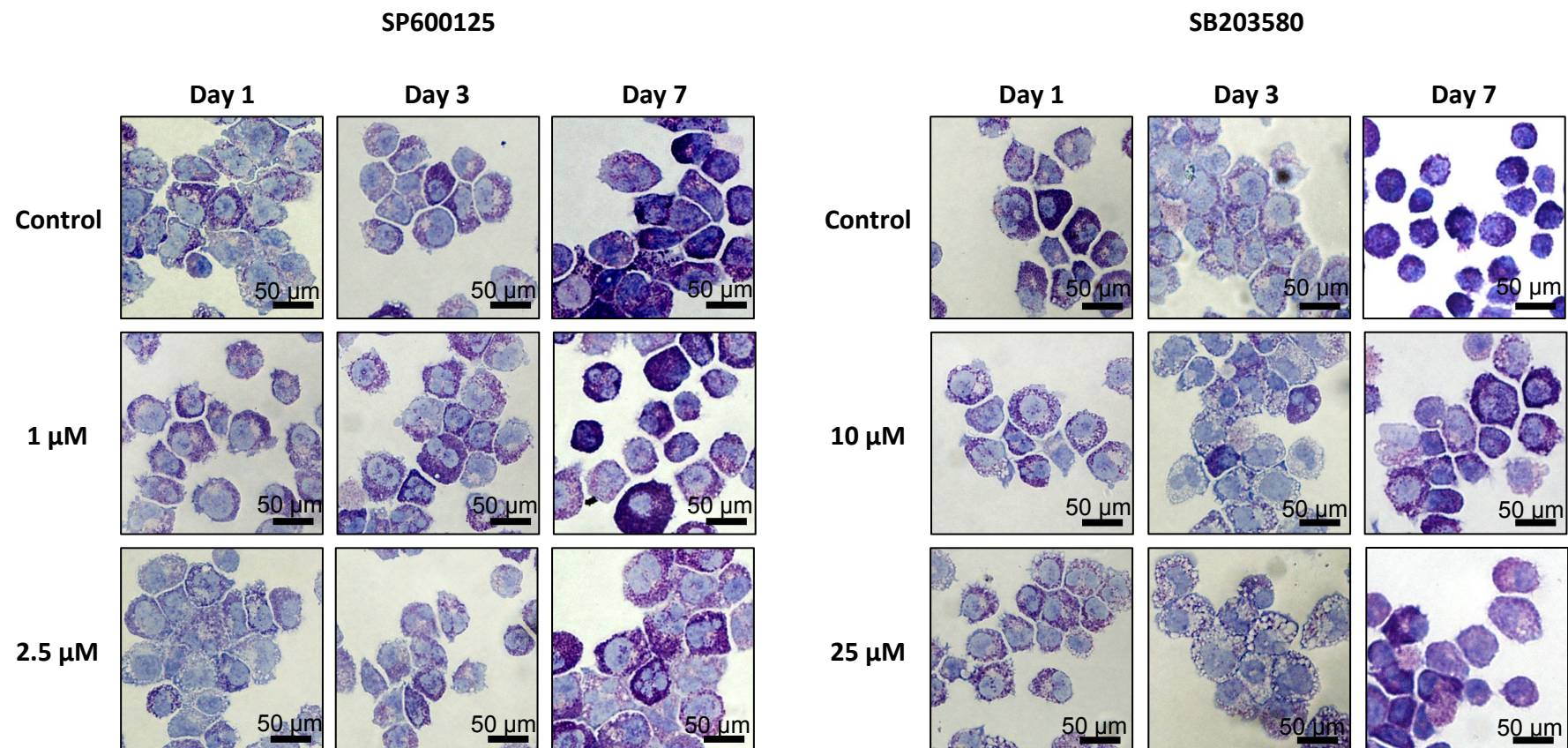

Suppl. Fig. 2

Supplement: Figure S2 — JNK or P38 mitogen-activated protein kinase (MAPK) inhibition does not enhance granule maturation in mast cells (MCs). Bone marrow-derived mast cells (MCs) (0.5–1.0 × 10^6^ cells/ml) were incubated with SP600125 (JNK MAPK inhibitor) or SB203580 (p38 MAPK inhibitor) at the concentrations and time periods indicated. After incubation with the respective inhibitor, cytospin slides were prepared and stained with May-Grünwald/Giemsa. The insets represent enlarged images of MCs with representative morphology. Note that neither of the inhibitors caused increased May-Grünwald/Giemsa staining intensity of granules. Original magnification: 40×. [file data_sheet_2.PDF]

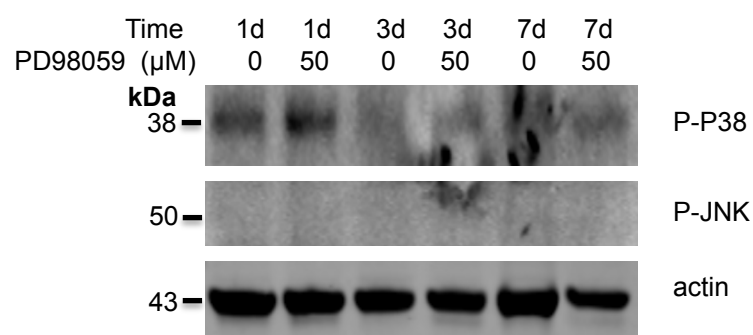

Suppl. Fig. 3

Supplement: Figure S3 — Mast cells (1 × 10^6^ cells) were left untreated or were treated with PD 98059 at the time periods and concentrations indicated. Cells were recovered and analyzed by western blotting for phosphorylation of P38 and JNK. Actin was used as loading control. [file data_sheet_3.PDF]

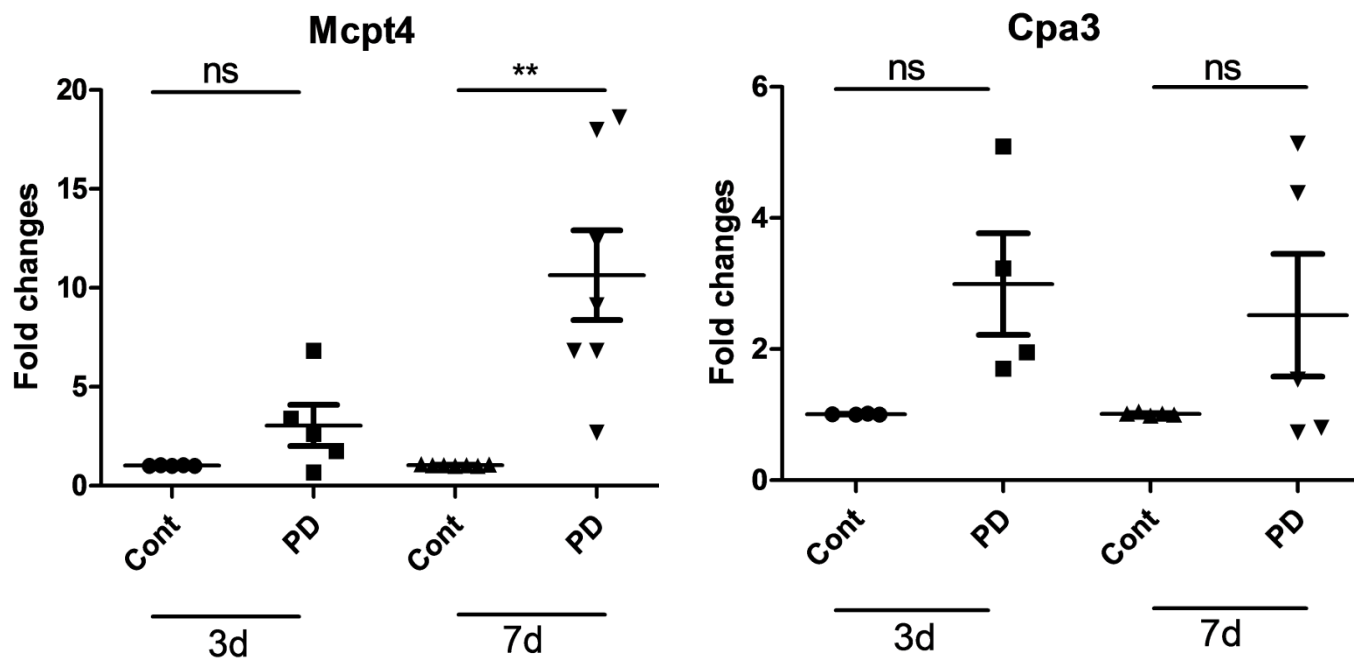

Suppl Fig. 4

Supplement: Figure S4 — Mast cells (0.5–1.0 × 10^6^ cells/ml) were incubated with PD98059 (50 µM) for the time periods indicated, followed by isolation of total RNA and quantification of mRNA encoding Mcpt4 and Cpa3 by qPCR (n ≥ 3). [file data_sheet_4.PDF]
